# Low Rate of Pandemic A/H1N1 2009 Influenza Infection and Lack of Severe Complication of Vaccination in Pregnant Women: A Prospective Cohort Study

**DOI:** 10.1371/journal.pone.0052303

**Published:** 2012-12-27

**Authors:** Odile Launay, Anne Krivine, Caroline Charlier, Van Truster, Vassilis Tsatsaris, Jacques Lepercq, Yves Ville, Carolyn Avenell, Thibaut Andrieu, Flore Rozenberg, Florence Artiguebielle, Jean-Marc Tréluyer, François Goffinet, Inserm COFLUPREG Study Group

**Affiliations:** 1 Université Paris Descartes, Paris, France; 2 Inserm, CIC BT505, Paris, France; 3 Assistance-Publique Hôpitaux de Paris (AP-HP), Hôpital Cochin, CIC de Vaccinologie Cochin-Pasteur, Paris, France; 4 AP-HP, Hôpital Cochin, Service de Virologie, Paris, France; 5 AP-HP, Hôpital Necker-Enfants Malades, Paris, France; 6 AP-HP, Hôpital Cochin, Maternité, Paris, France; 7 AP-HP, Hôpital Necker-Enfants Malades, Maternité, Paris, France; 8 Inserm U953, Recherche Epidémiologique en Santé Périnatale et Santé des Femmes et des Enfants, Université Pierre-et-Marie-Curie, Paris, France; 9 Inserm CIC P0901, Paris, France; Melbourne School of Population Health, Australia

## Abstract

**Background:**

In 2009, pregnant women were specifically targeted by a national vaccination campaign against pandemic A/H1N1 influenza virus. The objectives of the COFLUPREG study, initially set up to assess the incidence of serious forms of A/H1N1 influenza, were to assess the consequences of maternal vaccination on pregnancy outcomes and maternal seroprotection at delivery.

**Methods:**

Pregnant women, between 12 and 35 weeks of gestation, non vaccinated against A/H1N1 2009 influenza were randomly selected to be included in a prospective cohort study conducted in three maternity centers in Paris (France) during pandemic period. Blood samples were planned to assess hemagglutination inhibition (HI) antibody against A/H1N1 2009 influenza at inclusion and at delivery.

**Results:**

*Among the 877 pregnant women included in the study, 678 (77.3%) had serum samples both at inclusion and delivery, and 320 (36.5%) received pandemic A/H1N1 2009 influenza vaccine with a median interval between vaccination and delivery of 92 days (95% CI* 48–134). At delivery, the proportion of women with seroprotection (HI antibodies titers against A/H1N1 2009 influenza of 1∶40 or greater) was 69.9% in vaccinated women. Of the 422 non-vaccinated women with serological data, 11 (2.6%; 95%CI: 1.3–4.6) had laboratory documented A/H1N1 2009 influenza (1 with positive PCR and 10 with serological seroconversion). None of the 877 study’s women was hospitalized for flu. No difference on pregnancy outcomes was evidenced between vaccinated women, non-vaccinated women without seroconversion and non-vaccinated women with flu.

**Conclusion:**

Despite low vaccine coverage, incidence of pandemic flu was low in this cohort of pregnant women.No effect on pregnancy and delivery outcomes was evidenced after vaccination.

## Introduction

There is strong evidence that pregnant women and infants are at increased risk of severe illness following infection with influenza virus [1]. Hospitalization for respiratory illness related to seasonal influenza is more frequent in pregnant than in non pregnant women [2], [3], and the risk of death in pregnant women increased during influenza pandemics compared to non-pandemic years [4].

The emergence of A/H1N1 influenza infection in Mexico and in Australia in early 2009 raised further awareness and concern worldwide. In June 2009, World Health Organization raised the pandemic alert level to the highest level of 6 [5]. In August 2009, researchers from the Centers for Disease Control and Prevention reported that 6/45 (13%) patients who died from 2009 A/H1N1 influenza between mid-April and mid-June were pregnant women [6]. The disproportionately increased risk of mortality due to A/H1N1 2009 influenza infection in pregnant women was confirmed by the Centers for Disease Control and Prevention survey [6]. Pregnant women have been therefore designated as a top priority group to receive the pandemic A/H1N1 2009 influenza vaccine [7]–[11]. In France, the vaccination campaign was launched in November 2009; a single dose of a non-adjuvanted A/H1N1 2009 influenza vaccine was recommended for all pregnant women after the first trimester [11].

Most of available data are issued from retrospective studies and prospective cohort studies are still lacking to better understand how A/H1N1 2009 influenza pandemic affects pregnant women. Furthermore, whereas some studies have shown safety, immunogenicity and effectiveness of seasonal flu vaccination in pregnant women [4], [12], [13], additional data are still needed to assess the safety and efficacy of maternal vaccination during pandemic period.

In the context of the A/H1N1 2009 influenza pandemic, we planned a prospective study conducted in the general population of pregnant women to assess the incidence, the maternal-fetal impact of 2009 influenza pandemic, and the effectiveness and the safety of maternal vaccination. When it appeared that the pandemic level expected by public health services would be not achieved, the objectives of the study were redefined to assess: 1) the incidence of laboratory-documented influenza 2009 pandemic, 2) the effects of pandemic vaccination on pregnancy outcome and 3) the proportion of women with seroprotection against influenza 2009 A/H1N1 at delivery, both in vaccinated and in non-vaccinated women.

## Patients and Methods

### Pandemic A/H1N1 2009 Influenza and Vaccination in France

In France, a first wave of A/H1N1 2009 influenza infection was reported in July 2009. Detection of pandemic A/H1N1 2009 influenza viruses remained then sporadic until week 42 (October 12–18). In the second wave, influenza like illness (ILI) incidence peaked in week 49 (November 29-December 4) and fell below the epidemic threshold in the last week of the year [14]. Pandemic A/H1N1 2009 influenza vaccine was administered, free of charge, in centers dedicated to pandemic vaccination. A single dose of a non-adjuvanted A/H1N1 2009 influenza vaccine (Panenza®) was recommended for all pregnant women after the first trimester of pregnancy. On November 20, 2009, Panenza® was available and women in the second or third trimester of pregnancy asked to get vaccinated by receiving a letter from the National health insurance.

### Study Design

The COFLUPREG (COhort on FLU during PREGnancy) study was a prospective cohort study conducted on pregnant women in three tertiary maternity centers in Paris (France) during the pandemic A/H1N1 2009 influenza. Women were included from October 12, 2009 to February 3, 2010.

Pregnant women between 12 and 35 weeks of gestation were eligible to the study if they were aged ≥18 years and were speaking and understanding French. Main exclusion criteria were laboratory documented A/H1N1 2009 influenza during the last 6 months and vaccination against A/H1N1 2009 influenza before inclusion.

Pregnant women were randomly selected in order to obtain a representative sample of pregnant women followed in these maternity hospitals. The draw was made on the list of women to visit the following week in each of the maternity hospitals. The randomization numbers were established using tables of order for permutation and this procedure was stratified according to center. The sampling fraction was 1 in 4 and could theoretically include until 25 women a day for consultation across all three centers. The randomization plan and generated list were only known to study personnel not involved in clinical procedures. The selected women were contacted by phone one week before the scheduled date of the consultation to inform them of the study. If they were interested in participating, documents and written information were sent. The day of consultation, the women signed the informed consent and the data for inclusion were then filled using a specific case report form.

At inclusion in the study, the following data were collected: socio-demographic characteristics (mother age, geographic origin, lifestyle (single or couple), socio-professional category), medical factors (co-morbidity associated with a high-risk of occurrence of severe form of flu, flu symptoms since the beginning of pregnancy, seasonal flu vaccination in the previous 5 years, smoking), obstetrical characteristics (gestational age, gestity, parity, twin pregnancy, significant obstetrical history, current pregnancy complication) and factors associated with a higher risk of viral exposure and disease-spreading (number of children under 18 years of age at home, work in contact with children, healthcare workers and professional with contact with the public). Co-morbidity associated with a risk of occurrence of severe flu was defined by the presence of at least one of the following diseases: chronic lung disease (including asthma), severe cardiopathy, severe chronic nephropathy, severe neuropathy, severe myopathy, sickle-cell disease, diabetes mellitus, immunodeficiency, morbid obesity and alcoholism with chronic hepatopathy. Significant obstetrical history was defined as having at least one of the following events: late miscarriage (between 14^th^ and 21^th^ +6 days weeks of gestation), preterm delivery (between 22^th^ and 36^th^ +6 days weeks of gestation), and history of pre-eclampsia/gestational hypertension, intrauterine growth restriction, fetal malformation or fetal death. Current pregnancy complication was defined as having at least one of the following complications: placenta prævia, pyelonephritis, pre-eclampsia/gestational hypertension, gestational diabetes mellitus, suspicion of intrauterine growth restriction, fetal malformation, preterm labor and premature rupture of membranes (PROM).

All the included women were followed by doctors or midwifes with monthly visits until delivery. During each visit, information on the occurrence of fever or respiratory symptoms or documented A/H1N1 influenza infection and vaccination against A/H1N1 2009 influenza (participant verbal report) was prospectively collected in the case report form by a clinical research assistant dedicated to the study.

After inclusion in the study, women having fever, respiratory symptoms, or a contact with documented case of A/H1N1 influenza infection were asked to consult at the maternity as soon as possible. Women having an ILI defined as an oral temperature of more than 37.8°C with at least one influenza-like symptom (cough, sore throat, rhinorrhea, nasal obstruction) were asked to provide specimens of nasal and throat swabs for virology testing and blood sample for assessment of HI antibodies against A/H1N1 2009 influenza.

At delivery, maternal and perinatal outcome data were collected: maternal outcomes were onset of labor, mode of delivery, occurrence of fever during labor, and post partumhaemorrhage. Perinatal outcomes were fetal and neonatal death, gestational age at delivery, birth weight, Apgar score at 5 min, and transfer to neonatal intensive care unit.

Blood samples were planned for assessment of HI antibodies against A/H1N1 2009 influenza at inclusion and at delivery, and in case of ILI.

Written informed consent was obtained from each woman before enrolment. The protocol was conducted in accordance with the Declaration of Helsinki and French law for biomedical research and was approved by the “Ile-de-France 3” Ethics Committee (Paris, France). This study is registered with ClinicalTrials.gov: NCT01192737.

### Laboratory Methods

#### Hemagglutination inhibition antibodies against A/H1N1 2009 influenza

Immunological assays were performed in a centralized laboratory (Virology Laboratory, Cochin Hospital, Paris, France) in a blind way. The titer of antibodies against the vaccine strain was measured in all samples by hemagglutination-inhibition (HI) assay as described by the WHO Collaborating Centre for Influenza, Centres for Diseases Control, Atlanta, USA [15]. Serum samples were treated by enzymatic treatment to destroy nonspecific inhibitors. Sera were then tested in serial two-fold dilutions starting at 1∶10, all sera from a single patient being tested on the same plate. Hemagglutination was performed in a microtiter plate using human O rhesus negative erythrocytes and 4 units of A/California/7/2009 (H1N1v) vaccine as antigen (Panenza®). The sample titer was the highest dilution that completely inhibited hemagglutination. Negative samples were assigned a titer of 1∶5. The geometric mean HI antibody titers at each time point were used for the analyses.

Seroprotection rate was defined as the percentage of women with a HI titer of 1∶40 or greater, seroconversion rate as the percentage of women with a HI titer <1∶10 at inclusion and a titer of 1∶40 or greater at delivery, or showing a significant increase in antibody titer defined as a titer of 1∶10 or greater at inclusion and at least a fourfold increase in titer between inclusion and delivery [16]–[18].

#### Molecular detection of H1N1pdm09 pandemic influenza a virus

Nasopharyngeal secretions were collected by endonasal swabbing using flocked nylon swabs. H1N1pdm09 infection was diagnosedby real-time reverse transcription–PCR (RT-PCR) assay on a 7500 Real Time PCR System (Applied Biosystems, Foster City, CA) according to the protocol designed by the National Influenza Center Northern-France (Institut Pasteur, Paris, France) (http://www.sante.gouv.fr/IMG/pdf/Protocoles_CNR_03122009.pdf). PCRs were done locally.

### Statistical Analysis

A sample size of 2000 patients was initially planned to evaluate the incidence and the characteristics of A/H1N1 2009 influenza infection in the population of pregnant women. Indeed, with the initial hypotheses of an attack rate of A/H1N1 influenza up to 40% [19] in the absence of intervention, the inclusion of 2000 pregnant women in the cohort could allow the evaluation of about 800 cases of influenza infection. With an estimated frequency of severe forms requiring hospitalization of about 30%, about 130 of the 2000 women would have developed severe influenza [6], a number of cases enough to evaluate the incidence and the characteristics of A/H1N1 2009 influenza infection in pregnant women. When it appeared for epidemiological reasons (both lower attack rate and frequency of severe forms) that the objectives of the study could not be achieved, the H1N1 independent advisory board of the “Institut de Microbiologie et des Maladies Infectieuses” (IMMI) decided to stop inclusion in February 2010 after 919 inclusions. The modified endpoints were: effects of pandemic vaccination on pregnancy outcomes (gestational age at delivery, mode of delivery, mean birth weight, Apgar score, neonatal outcome) and the standard HI endpoints (seroprotection rate, geometric mean titers, seroconversion ratewith 95% confidence intervals [CI]) for immunogenicity at delivery, both for vaccinated and not vaccinated pregnant women.

Chi-square test or Fisher’s exact test (in cases of low number of data) were used for comparisons of qualitative variables. Kruskal-Wallis test was used for comparison of quantitative variables. A p-value <0.05 was considered significant.

For proportions, exact binomial 95% CI were calculated. For geometric mean titers, 95% CI were computed by taking the exponent (log_10_) of the lower and upper limits of the 95% CI of the log_10_-transformed titers.

## Results

### Study Patients

**Figure 1:**
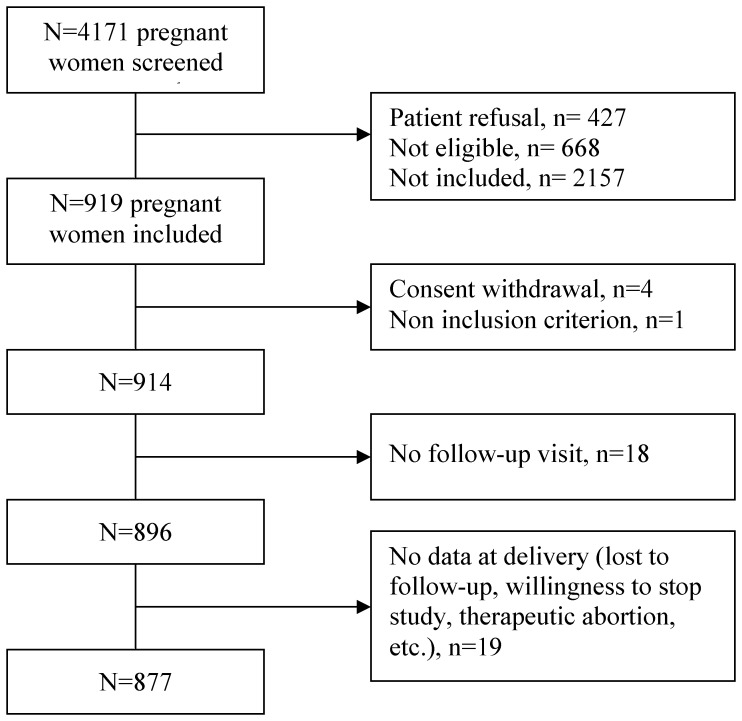
Disposition of pregnant women in the COFLUPREG cohort.

A total of 4171 women were screened, among whom 427 refused to participate, 668 were ineligible, and 2157 were not included.Women were included from October 12, 2009 to February 3, 2010; first delivery occurred in November 2009 and last delivery in August 2010. Among the 919 pregnant women included, 4 withdrew their consent and 1 had exclusion criteria; 37 women (4%) were excluded of analysis due to loss of follow up (i.e. women who gave birth in another hospital and with less than 3 follow-up visits) (Figure 1). No difference in maternal baseline characteristics was evidenced between the 877 pregnant women included in the study and the 37 pregnant women excluded of the analysis.

The demographic profiles and the clinical characteristics of the 877 women of the cohort are described in Table 1. Among them, 507 (57.8%) were included with gestational age between 12 and 22 weeks, 203 (23.1%) between 22 and 28 weeks, and 167 (19.0%) between 28 and 35 weeks. Blood sample was available at baseline for 825 (94.1%) of those 877 women; 43 (5.2%) had HI antibodies against 2009 A/H1N1 influenza with titers of 1∶40 or greater.

**Table 1:** Participant characteristics.

Characteristics	N (%) Total = 877	
Centers		
Center A	214 (24.4)	
Center B	433 (49.4)	
Center C	230 (26.2)	
Maternal age at inclusion, years		
18–24	39 (4.5)	
25–34	544 (62.0)	
≥35	294 (33.5)	
Body mass index, kg/m^2^		
<18.5	67 (7.7)	
18.5–25	639 (72.9)	
>25	170 (19.4)	
Geographic origin		
Metropolitan France	582 (66.3)	
Overseas France	16 (1.8)	
Europe	52 (5.9)	
North Africa	90 (10.3)	
Sub-Saharan Africa	49 (5.6)	
Asia	26 (3.0)	
Other	62 (7.1)	
Single	57 (6.5)	
Number of children under 18 years at home		
0	424 (48.3)	
1	312 (35.6)	
>2	141 (16.1)	
Job associated with a higher risk of viral exposure	467 (53.2)	
Work in contact with the children	87 (9.9)	
Healthcare worker	88 (10.0)	
Professionals in contact with the public	401 (45.7)	
Seasonal flu vaccination in the previous 5 years	131 (14.9)	
Primiparous	415 (47.3)	
Gestational age (weeks) at inclusion		
<22	507 (57.8)	
[22]–[28]	203 (23.1)	
>28	167 (19.0)	

### Follow up

Between inclusion and delivery, only three women benefited of an additional visit for ILI: one of them at 19 weeks of gestation had positive 2009 A/H1N1 influenza PCR, one was PCR-negative, and no PCR was done for the third one. The woman with confirmed A/H1N1 influenza received oseltamivir and delivery occurred at term without complication for mother and infant. The two other women did not receive oseltamivir and delivered at term without complication for mother and infant.

A total of 55 women reported ILI, among whom only 3 benefited of an additional visit at the maternity, and 72 women reported contact with a H1N1 case, among whom 24 had a nasal swab with negative result.

A total of 39 women (including the woman previously mentioned with positive PCR) (4.5%) received oseltamivir without additional visit or PCR (20 for ILI, and 19 for preventive reasons), including 25 (64.1%) women who were not vaccinated against A/H1N1 2009 influenza.

None of the 877 women was hospitalized for influenza.

Three hundred and twenty (36.5%) women were vaccinated against pandemic A/H1N1 2009 influenza between inclusion and delivery. Median gestational age at vaccination was 23.6 weeks (95% CI: 18.7–30.6) and median interval between vaccination and delivery was 92 days (95% CI: 48–134).

### Immune Status Against Pandemic A/H1N1 2009 Influenza

Serum samples both at inclusion and delivery were available for 678 (77.3%) women, of whom 256 (37.8%) had received A/H1N1 2009 influenza vaccine.

At inclusion, 13 (5.1%) of vaccinated women and 19 (4.5%) of non-vaccinated women had HI antibodies against A/H1N1 2009 influenza strain with titers of 1∶40 or greater (Table 2). At delivery, the seroprotection rate was 30.3% (95% CI: 26.8–33.9): 69.9% in vaccinated women, and 6.2% (95% CI: 4.1–8.9) in non-vaccinated women.

**Table 2:** Humoral immunity against pandemic A/H1N1 2009 influenza in vaccinated and non-vaccinated pregnant women at baseline and delivery (n = 678).

	2009 A/H1N1 influenza vaccinated pregnant women N = 256	Non-vaccinated pregnant women N = 422
**At inclusion**		
Geometric mean titer [95% CI]	7.3 [6.7–8.0]	6.7 [6.3–7.1]
Number (%) of women with HI titers >1∶40 [95% CI]	13 (5.1) [2.7–8.5]	19 (4.5) [2.7–7.0]
**At delivery**		
Geometric mean titer [95% CI]	49.8 [43.0–57.7]	7.3 [6.8–7.8]
Number (%) of women with HI titers >1∶40 [95% CI]	179 (69.9) [63.9–75.5]	26 (6.2) [4.1–8.9]
Seroconversion rate§, Number (%) of women [95% CI]	171 (66.8) [60.1–72.5]	10 (2.3) [1.0–4.0]

§ Seroconversion rate is given as the percentage of women with a HI titer <1∶10 at inclusion and a titer of 1∶40 or greater at delivery, or showing a significant increase in antibody titer defined as a titer of 1∶10 or greater at inclusion and at least a fourfold increase in titers between inclusion and delivery.

Ten (2.3%) of the 422 women who did not receive the vaccine seroconverted between inclusion and delivery. None of them reported both fever and at least one flu symptom; one had isolated fever, two had isolated respiratory symptoms without fever, and six did not report any flu symptoms. None of them received oseltamivir.

Finally, flu was laboratory-documented in 11 women among the 422 non-vaccinated women with serological data (1 with positive PCR and 10 with serological seroconversion) (rate 2.6 per 100 pregnant women [95% CI: 1.3–4.6]) and no severe flu occurred.

### Consequences of A/H1N1 2009 Influenza Vaccination on Pregnancy Outcomes

There was no significant difference on pregnancy (onset of labour, mode of delivery,gestational age at delivery) and perinatal outcomes (birth weight, Apgar score, and transfer to neonatal care unit) between women who received A/H1N1 2009 influenza vaccine and women non-vaccinated (Table 3). Determinants of non vaccination were studied in the cohort and previously published [20]. In a multivariate logistic regression, immigrant women and those having a low socio-economic status were independent factors of non vaccination. We compared pregnancy and perinatal outcomes in vaccinated and non vaccinated women according to different categories of “immigrant women” and “socio-economic status”. No significant differences were evidenced between the two groups (data not shown).No difference on pregnancy and perinatal outcomes was evidenced between vaccinated women, non-vaccinated women without seroconversion, and women with virologically confirmed influenza or who seroconverted without vaccination, and between women who received oseltamivir and those who did not receive oseltamivir (data not shown).

**Table 3:** Consequences of pandemic A/H1N1 2009 influenza vaccination on pregnancy outcomes.

	A/H1N1 2009 influenza vaccinated pregnant women	Non-vaccinated pregnant women	p-value
	n = 320	n = 557	
Gestational age (weeks) at delivery,edian (IQR)	39.5 (38.6–40.9)	39.4 (38.7–40.6)	-
Delivery <37 weeks, n (%)	22 (6.9)	41 (7.4)	0.80
Onset of labour, n (%)			
Spontaneous	210 (66.7)	367 (66.4)	
Induction	71 (22.5)	130 (23.5)	0.92
Caesarean	34 (10.8)	56 (10.1)	
Fever during labour, n (%)	26 (8.1)	53 (9.5)	0.51
Mode of delivery, n (%)			
Spontaneous vaginal delivery	210 (66.7)	379 (68.3)	
Instrumental vaginal delivery	34 (10.8)	47 (8.5)	0.53
Caesarean section	71 (22.5)	129 (23.2)	
Delivery hemorrhage, n (%)			
None	239 (90.5)	432 (90.9)	
<1 liter	23 (8.7)	35 (7.4)	-
>1 liter	2 (0.8)	8 (1.7)	
Birth weight, g			
Mean	3296.1	3282.0	
<2500, n (%)	22 (6.9)	33 (6.0)	
[2500–4000[, n (%)	273 (85.9)	485 (87.2)	0.81
≥4000, n (%)	23 (7.2)	38 (6.8)	
Apgar score <7 at 5 min, n (%)	1 (0.3)	5 (0.9)	0.42†
Infants outcome			
Alive at birth	317 (99.7)	552 (99.3)	
Dead before labour	1 (0.3)	3 (0.5)	-
Dead during labour	0 (0.0)	1 (0.2)	
Transfer to neonatal intensive care unit‡	31 (9.8)	61 (11.1)	0.58
Pregnancy loss	1 (0.3)	3 (0.5)	0.24
Fetal malformation	4 (1.25)	3 (0.5)	0.26
Pre eclampsia	1 (0.3)	2 (0.3)	1

† Fisher’s exact test;

‡ First infant for multiple birth.

## Discussion

In this cohort of pregnant women conducted during the H1N1 2009 pandemic, the number of laboratory-documented influenza infections remained low despite low vaccine coverage (36.5%): only one woman had PCR-confirmed A/H1N1 influenza and 10 non-vaccinated women seroconverted between inclusion and delivery; no serious case of influenza and no hospitalization for influenza were reported. Of note, the low level of influenza infection (rate of 2.6 per 100 pregnant women) is reliable since both PCR and serological data were combined for diagnosis.

It could be suggested that the low rate of influenza infection in our cohort was related to the willingness of women to participate to the study with a selection of women understanding preventive measures to avoid flu infection. However, vaccination rate (36.5%), although rather low, was close to the coverage rate in general population of French pregnant women (29.3%) [21] and we showed previously that vaccine coverage was not higher in women with higher risk of exposure to the virus [20]. Other effective ways to reduce the transmission of influenza virus including hygiene habits could have play some role in this cohort population aware of the issues related to A/H1N1 influenza during pregnancy and keener to avoid infection than the general population.

We did not evidence an impact of A/H1N1 influenza infection on maternal and perinatal outcomes but only few women experienced flu. The pregnancy outcomes did not appear to be seriously affected by pandemic and were comparable with those of the French perinatal survey during non pandemic periods [21]. In the national French registry created to screen pregnant women with laboratory-confirmed A/H1N1 2009 influenza, the morbidity and ICU management was increased mainly in at-risk patients [22]; maternal mortality remained low and was lower than mortality rates observed in other countries [23], [24]. The UK study of 256 pregnant women admitted to hospital with confirmed A/H1N1 influenza evidenced a significantly high risk of poor pregnancy outcome with higher perinatal mortality in infants born from infected vs. uninfected women (39/1000 vs. 7/1000 births, respectively) [25]. The low number of laboratory-documented influenza infections in our cohort does not allow extrapolating our results on maternal and perinatal outcomes. However, the design of our study is adapted to assess the incidence of flu in this population of pregnant women and the impact on less severe pregnancy outcomes. The differences of results between our study and others could be also explained by the characteristics of the population, health system and pandemic characteristics.

When it appeared that the infection rate of the pandemic was less than expected, the inclusions in the study were stopped but COFLUPREG cohort was nevertheless pursued since there was an opportunity to assess immunogenicity, safety and consequences of vaccination on outcomes of pregnancies. Indeed, vaccine safety is a special concern in pregnant women. Previous studies suggested that inactivated seasonal influenza vaccines were safe during pregnancy [26]–[31]. Data were lacking in pregnant women for the pandemic A/H1N1 2009 influenza vaccine, especially studies with comparative data on pregnancy outcome between vaccinated and non-vaccinated women. A French study in 107 pregnant women who received one dose of non-adjuvanted pandemic A/H1N1 2009 influenza vaccine between 22 and 32 weeks of gestation did not evidence adverse events of special interest [32]. The prospective study of Tavares et al with AS03-adjuvanted pandemic A/H1N1 2009 influenza vaccine in 267 pregnant women did not evidence an increase of the risk of adverse pregnancy outcomes (spontaneous abortion, congenital abnormalities, preterm delivery, low birth weight neonates or maternal complications) [33]. In a large cohort study conducted in Denmark among 54 585 pregnant women (7062 vaccinated women), no evidence of an increased risk of fetal death associated with exposure to an adjuvanted pandemic A/H1N1 2009 influenza vaccine during pregnancy was found [34]. In the present study, we confirm the safety of one injection of the non-adjuvanted A/H1N1 2009 influenza vaccine. Indeed, among the 320 pregnant women who were vaccinated, no significant difference on maternal and perinatal outcomes was observed in comparison with the group of 557 pregnant women who were not vaccinated.

Among vaccinated women, the seroprotection rate (defined as titers above 1∶40) at delivery was only 69.9%. In the PREFLUVAC study, performed with the same vaccine at the same period, the women were vaccinated between the 22^th^ and 26^th^ weeks of pregnancy and 92% of them achieved seroprotection at delivery; the median duration between vaccination and delivery was 12 weeks [32]. The COFLUPREG study was not designed as a vaccine trial and was performed in naturalistic real life conditions. Therefore, the follow-up of each woman varied widely according to time of delivery, between 2 and 8 months after vaccination. This could explain to some extent the lower seroprotection rate in the COFLUPREG study among vaccinated women. The interlaboratory variability of HI method has also been previously reported and could also explain why seroprotection rate at delivery here appeared lower than expected [35].

Our study has some limits. First, due to the change of the primary objective and early arrest of inclusion, the study was not powered for assessment of rare serious events related to vaccination. Second, the study was performed in three clinical wards in academic hospitals in Paris and consequently the cohort could be not representative of the French population of pregnant women. Third, the groups of vaccinated and non-vaccinated pregnant women were not randomized. Therefore, it is possible that vaccinated women had not the same initial risk of pregnancy complications than non-vaccinated women. Indeed, we previously showed that the rate of coverage in the same cohort was low in immigrant women and women with low economic status and these conditions could be associated with poor pregnancy outcomes [20].

In conclusion, despite low vaccine coverage, incidence of pandemic flu was very low in this cohort of pregnant women. No effect on pregnancy and delivery outcomes was evidenced after vaccination. However, seroprotection rate at delivery appeared lower than expected in vaccinated women.
